# Supplementary material for: Deficiency in the cell-adhesion molecule dscaml1 impairs hypothalamic CRH neuron development and perturbs normal neuroendocrine stress axis function
Source: Front Cell Dev Biol. 2023 Feb 16;11:1113675. doi: 10.3389/fcell.2023.1113675 (PMC9978177; doi:10.3389/fcell.2023.1113675)
Supplement: Supplementary file 3 [file Presentation1.pdf]

Ma et al. 2023.

Supplementary Figures and Table

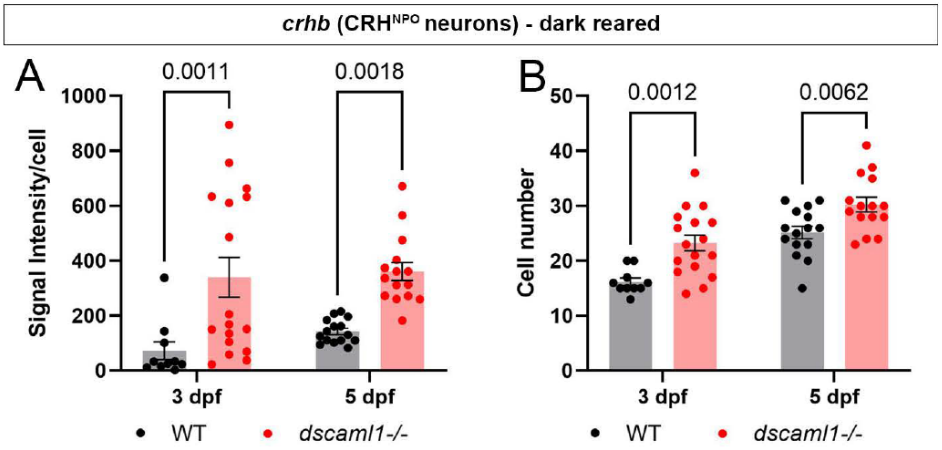

Supplementary Fig. S1. CRH^{NPO} neuron development is impaired in the absence of ambient light (dark reared). Quantification of CRH^{NPO} neurons, labeled by *crhb* FISH. Graphs show the signal intensity per cell (A) and cell number (B). Multiple-comparison corrected *p* values are as shown. WT: n=10 (3 dpf), 15 (5 dpf). *dscaml1-/-*: n=17 (3 dpf), 15 (5 dpf)

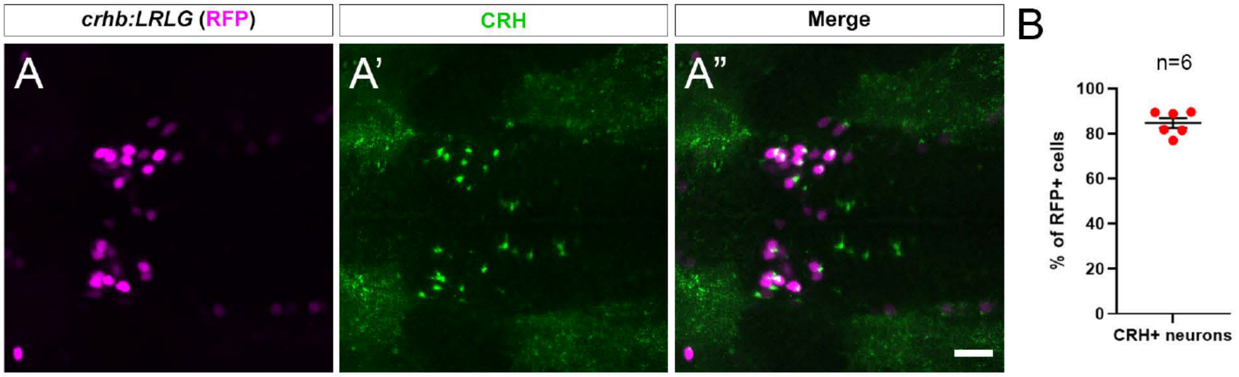

Supplementary Fig. S2. The *crhb:LRLG* transgenic line labels CRH-expressing neurons. (A-A") Most RFP+ neurons (magenta, anti-RFP) express the CRH protein (green, anti-CRH). (B) Percentage of RFP+ neurons that express CRH. Mean, standard error, and sample size are shown. The scale bar is 20 μm.

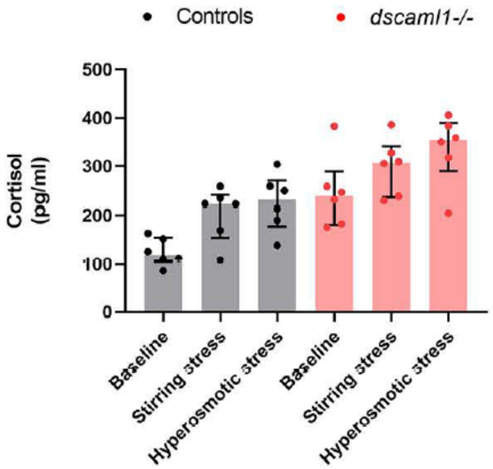

Supplementary Fig. S3. Non-normalized baseline and stress-induced cortisol profile for 5 dpf larvae at ZT6-8. Same measurements as Fig. 5B, but without normalization. For each sample (dot), cortisol was extracted from a pool of 30 animals. n=6 for all groups. Controls in black and *dscaml1-/-* animals in red. Median and interquartile range are shown.

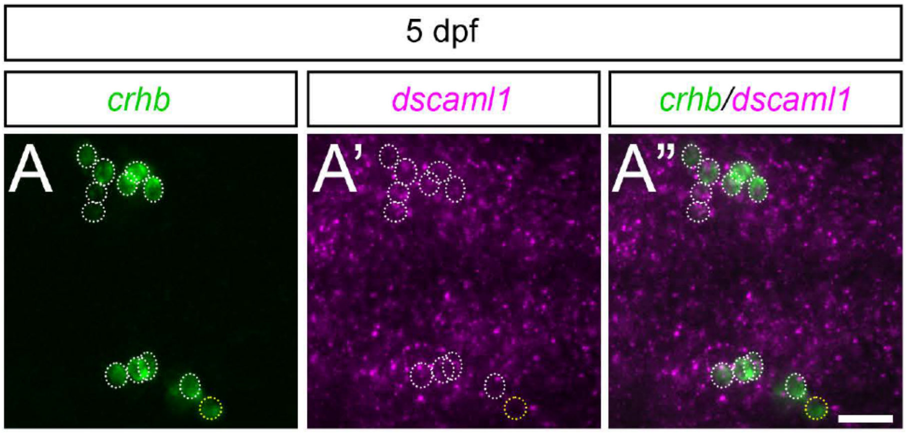

Supplementary Fig. S4. *dscaml1* is expressed in CRH^{NPO} neurons during larval development. Double FISH with *crhb* (green, A) and *dscaml1* (magenta, A') RNA probes at 5 dpf. Merged image is shown in A". Dotted circles mark the outline of *crhb*+ cells that express *dscaml1* (white) or do not express *dscaml1* (yellow). The scale bar is 20 μm.

**CRH^{NPO} neurons (normal day/night cycle) (Fig. 2E-F)**			
***crhb* signal intensity per cell**	**F (DFn, DFd)**	**P value**	**Significant?**
Interaction	F (2, 71) = 7.649	P=0.0010	Yes
Developmental stage	F (2, 71) = 10.48	P=0.0001	Yes
Genotype	F (1, 71) = 43.31	P<0.0001	Yes
**Cell number**	**F (DFn, DFd)**	**P value**	**Significant?**
Interaction	F (2, 71) = 9.102	P=0.0003	Yes
Developmental stage	F (2, 71) = 75.67	P<0.0001	Yes
Genotype	F (1, 71) = 4.865	P=0.0306	Yes

**CRH^{NPO} neurons (dark reared) (Supp. Fig. S1)**			
***crhb* signal intensity per cell**	**F (DFn, DFd)**	**P value**	**Significant?**
Interaction	F (1, 53) = 0.2373	P=0.6282	No
Developmental stage	F (1, 53) = 0.8534	P=0.3598	No
Genotype	F (1, 53) = 24.36	P<0.0001	Yes
**Cell number**	**F (DFn, DFd)**	**P value**	**Significant?**
Interaction	F (1, 53) = 0.5935	P=0.4445	No
Developmental stage	F (1, 53) = 36.83	P<0.0001	Yes
Genotype	F (1, 53) = 21.35	P<0.0001	Yes

***crhb:LRLG* fluorescent positive (Fig. 5G)**			
**Cell number/hemisphere**	**F (DFn, DFd)**	**P value**	**Significant?**
Interaction	F (1, 45) = 0.5128	P=0.4776	No
Developmental stage	F (1, 45) = 50.97	P<0.0001	Yes
Genotype	F (1, 45) = 7.544	P=0.0086	Yes

**Dex treatment (Fig. 7C)**			
***crhb* signal intensity per cell**	**F (DFn, DFd)**	**P value**	**Significant?**
Interaction	F (1, 35) = 8.210	P=0.0070	Yes
Treatment	F (1, 35) = 59.48	P<0.0001	Yes
Genotype	F (1, 35) = 11.91	P=0.0015	Yes

Supplementary Table I
